# An eastward-propagating coupled mode amplifying the early growth of extreme El Niño events

**DOI:** 10.1126/sciadv.aee0444

**Published:** 2026-09-23

**Authors:** Yu Liang, Shang-Ping Xie, Alexey Fedorov, Qihua Peng, Ayumu Miyamoto

**Affiliations:** ^1^ Scripps Institution of Oceanography, University of California San Diego, La Jolla, CA, USA.; ^2^ Thrust of Earth, Ocean and Atmospheric Sciences, The Hong Kong University of Science and Technology (Guangzhou), Guangzhou, China.; ^3^ Advanced Institute for Marine Ecosystem Change, Tohoku University, Sendai, Japan.; ^4^ Department of Earth and Planetary Sciences, Yale University, New Haven, CT, USA.; ^5^ LOCEAN/IPSL, Sorbonne University, Paris, France.; ^6^ School of Atmospheric Sciences, Nanjing University, Nanjing, China.

## Abstract

Extreme El Niño events are among the most consequential climate phenomena, yet their early prediction remains challenging. Here, by analyzing the 1982/1983, 1997/1998, and 2015/2016 events together with a large ensemble of climate model simulations, we identify an unusually early and reproducible ocean-atmosphere precursor to extreme El Niño: a slow, eastward-propagating coupled mode that develops in the western-central equatorial Pacific during boreal winter and spring. This mode features warm sea surface temperature anomalies coupled with sustained westerly wind anomalies. These westerly wind anomalies drive the equatorward transport of warm water, rapidly increasing equatorial ocean heat content and priming extreme El Niño growth in the following summer and fall. Ensemble hindcasts further reveal that East Asian cold surges and the North Pacific Oscillation can stochastically excite this equatorial mode, enabling skillful forecasts of extreme events as early as February and helping to overcome the spring predictability barrier.

## INTRODUCTION

The El Niño–Southern Oscillation (ENSO) is the dominant mode of interannual climate variability, alternating between El Niño and La Niña phases with periodicity of 2 to 7 years (*1*, *2*). Rooted in the tropical Pacific, its influence extends worldwide, shaping rainfall, droughts, and temperature extremes that disrupt ecosystems and societies (*3*). The most extreme El Niño events (1982/1983, 1997/1998, and 2015/2016) have produced unparalleled impacts, ranging from severe floods in Peru and the Yangtze River basin (*2*, *4*) to droughts across the Maritime Continent and the Amazon basin (*5*–*7*), widespread coral bleaching (*8*), and sharp rises in global mean surface temperature (*9*, *10*). Despite these outsized impacts, the physical mechanisms that trigger such extreme El Niño events remain debated, limiting predictive capability and preparedness (*11*, *12*).

The prevailing paradigm attributes the onset of extreme El Niño events to the interplay between recharged equatorial upper-ocean heat content (OHC) and episodic westerly wind bursts (WWBs) (*13*–*17*). Enhanced OHC early in the year typically precedes peak sea surface temperature (SST) anomalies and has long been viewed as the principal precondition for El Niño growth (*18*). This elevated OHC is conventionally attributed to the slow recharge process in response to preceding easterly wind anomalies (*13*, *18*, *19*), whereas WWBs are treated as stochastic disturbances (*20*–*25*) that can trigger rapid warming when they happen to occur over a precharged equatorial Pacific (*15*, *16*). However, recent studies suggest that westerly wind anomalies can also enhance OHC by inducing equatorward Ekman transport (*26*–*28*). This westerly wind–induced OHC increase occurs as a fast process related to the excitation of downwelling Kelvin waves, followed by a longer-term discharge linked to off-equatorial Rossby wave adjustment (*26*, *28*, *29*). In contrast, the recharge process associated with easterly anomalies operates on a timescale of about a year or longer (*28*). Yet, the exact physical processes in building OHC and initiating extreme El Niño events remain unresolved.

Here, by analyzing the three historical extreme El Niño events and their counterparts in a climate model simulation, we identify a growing, deterministic coupled mode that is critical for building equatorial OHC during boreal winter-spring and for the onset of extreme events. This mode is related to the “SST mode” in the eastern Pacific identified in early theoretical ENSO studies (*30*–*32*). It features slow eastward-propagating warm SST anomalies in the western-central equatorial Pacific from November(−1) to May(0) (Materials and Methods), accompanied by equatorial westerly wind anomalies. Such SST anomalies have previously been interpreted as arising from the interaction between state-dependent WWBs and the eastern edge of the warm pool: WWBs drive eastward extension of the warm pool through zonal advection, and the extended eastern edge in turn favors further WWB activity (*33*–*37*). These westerly wind anomalies can drive a rapid increase in OHC, which, superimposed on an initially recharged state, preconditions the subsequent development of extreme El Niño events. Ensemble hindcast experiments further show that stochastic extratropical forcings can selectively excite this equatorial coupled mode, enabling the three historical extreme El Niño events to partially overcome the spring predictability barrier (*12*).

## RESULTS

### A coupled equatorial mode during extreme El Niño onset

**Fig. 1.**
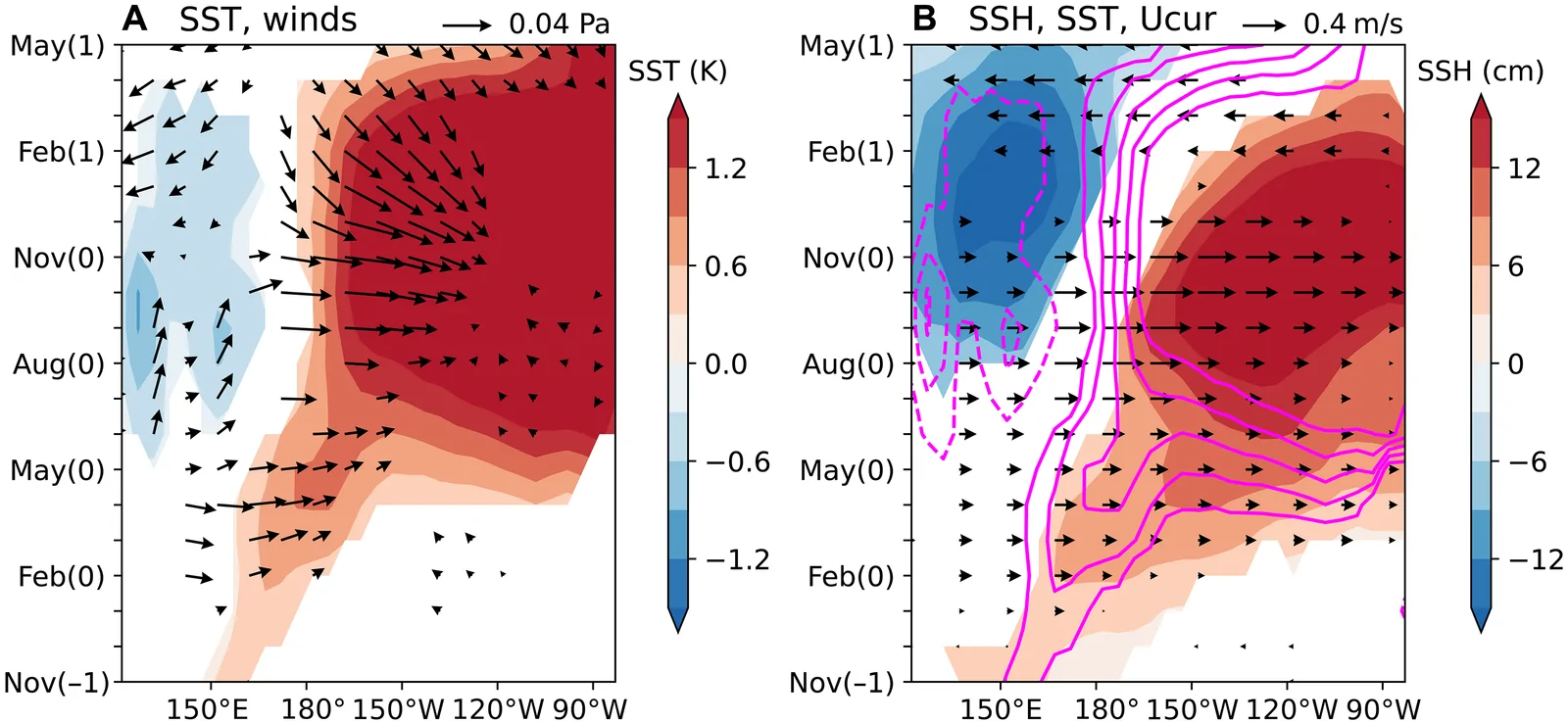
Spatiotemporal evolution of the three observed extreme El Niño events. (**A**) Hovmöller diagram of SST anomalies (shading) and wind stress anomalies (arrows), composited for the 1982/1983, 1997/1998, and 2015/2016 events. Unless otherwise noted, all subsequent results are based on this three-event composite. Notice an amplifying, eastward-propagating warm SST anomaly west of 160°W from November(−1) to May(0), preceding the rapid El Niño growth in the central-eastern equatorial Pacific. (**B**) Same as in (A) but for sea surface height anomalies (SSH; shading), SST anomalies (contours), and zonal current speed anomalies averaged over the upper 50 m (arrows). Anomalies are averaged within the equatorial band (5°N to 5°S) and a 3-month running mean is applied. Only signals with the same sign across all three events are retained.

During the onset of the three historical extreme El Niño events, despite different initial ocean states, a warm SST anomaly consistently emerges in the western-central equatorial Pacific from November(−1) to May(0), accompanied by westerly wind anomalies to its west and over its core (Fig. 1A and fig. S1, A to C; Materials and Methods). This anomaly precedes the major warming in the central-eastern equatorial Pacific, grows rapidly to ∼1 K by April to May(0), and slowly propagates eastward at ∼5° longitude per month. By contrast, El Niño events lacking this western-central Pacific precursor (Materials and Methods) only achieve moderate warming [∼1 K in the Niño 3.4 region by December(0); fig. S2], indicating an important role of this precursor in fueling extremes. A weak eastward-propagating SST signal can also be detected in these weaker El Niño events; however, it emerges later, around April(0), and remains substantially smaller in amplitude.

WWBs, defined relative to the daily climatology (*20*, *22*, *34*, *38*), often intensify at the early stage of extreme El Niño events (*14*, *15*, *17*, *35*, *39*). Atmospheric model experiments (Materials and Methods) show that in the three extreme cases, ∼80% of the equatorial westerly wind anomalies between February(0) and May(0) are forced by the underlying SST anomalies (fig. S3), consistent with findings for the 1997/1998 event (*40*). Thus, rather than acting as stochastic forcings, springtime WWBs during the onset of the three extreme events (*14*, *15*, *17*, *35*, *39*) predominantly reflect a deterministic wind response dynamically coupled to the growing SST anomaly, superimposed on high-frequency wind variability. It is this low-frequency deterministic component that is important for ENSO growth (*34*, *41*–*43*).

The westerly wind anomalies deepen the equatorial thermocline by generating downwelling Kelvin waves (*15*, *39*), manifested as elevated sea surface height (SSH) and anomalous eastward surface currents that propagate eastward (Fig. 1B). As will be shown later, westerly wind–induced off-equatorial upwelling Rossby waves also contribute to the anomalous eastward currents in the western-central equatorial Pacific. The resulting anomalous zonal advection is the main mechanism for the growth of the western-central equatorial Pacific SST anomaly based on a mixed layer heat budget analysis (Materials and Methods; fig. S4), consistent with previous studies (*36*, *44*). From an energetics perspective, the collocation of surface westerly wind anomalies with eastward surface current anomalies (Fig. 1 and fig. S5) supports the unstable growth of this coupled mode (*45*).

Early theoretical ENSO studies suggested the existence of an eastward-propagating “SST mode” in the eastern equatorial Pacific (*30*–*32*), in which zonal advection of the mean temperature gradient contributes to the mode growth and thermocline adjustment causes eastward propagation. In the western-central equatorial Pacific, however, the thermocline feedback is weak. Instead, we find that reduced net surface shortwave flux west of the SST anomaly and warm advection to its east facilitates its eastward propagation (fig. S4).

Meanwhile, the deepened thermocline, together with the anomalous eastward surface currents, causes warming in the central-eastern equatorial Pacific, which already reaches ∼1 K by May(0) (Fig. 1B and fig. S5F). This sets the stage for Bjerknes feedback in the following summer and fall (*46*), priming further growth of extreme El Niño events, as will be discussed in more detail.

### Evidence from the CESM2 Large Ensemble

**Fig. 2.**
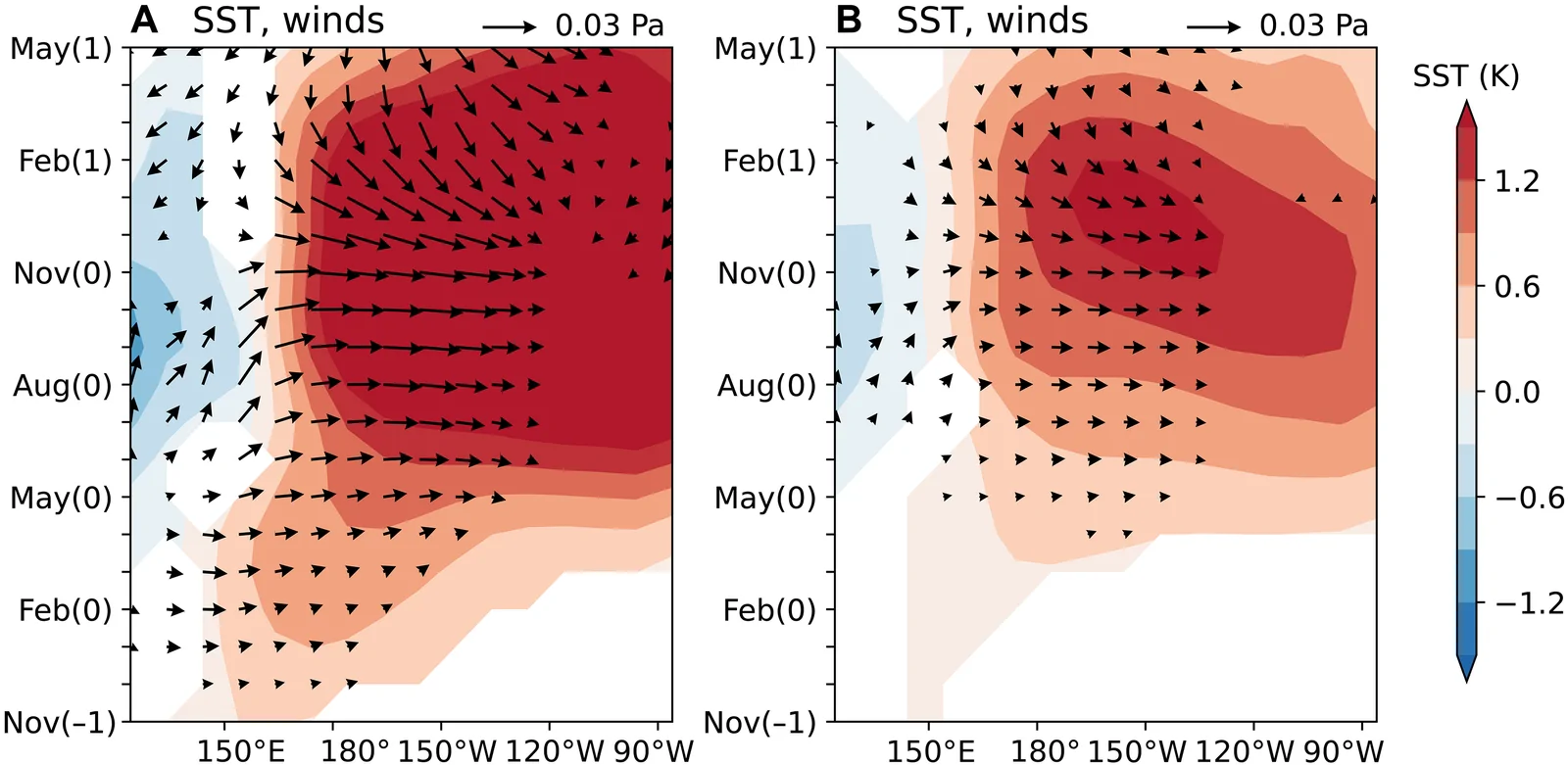
Composites of extreme and moderate El Niño events in the CESM2 Large Ensemble. (**A**) Hovmöller diagram of SST anomalies (shading) and surface wind stress anomalies (arrows), composited for 34 extreme El Niño events from 10 ensemble members during 1980 to 2019. (**B**) As in (A), but for 98 moderate El Niño events. Anomalies are averaged in the equatorial band (5°N to 5°S) and a 3-month running mean is applied. Only signals statistically significant at the 95% confidence level are shown. The precursory warm SST signal in the western-central equatorial Pacific during boreal winter-spring before El Niño is markedly weaker in (B) than in (A).

The role of the eastward-propagating warm SST anomaly in fueling extreme El Niño events is further supported by CESM2 Large Ensemble experiments (see Materials and Methods). By compositing 34 extreme El Niño events from 10 ensemble members over the period 1980 to 2019, we identify statistically significant warm SST anomalies in the western-central equatorial Pacific that propagate eastward during the event onset (Fig. 2A), closely resembling the evolution observed during the three historical extreme events. Among these 34 extreme events, 27 (∼80%) are preceded by this warm SST precursor (see Materials and Methods). In contrast, within the same 10 ensemble members, the 98 moderate El Niño events are preceded by a substantially weaker precursory warm SST signal in the western-central equatorial Pacific (Fig. 2B).

### Subtropical linkage

**Fig. 3.**
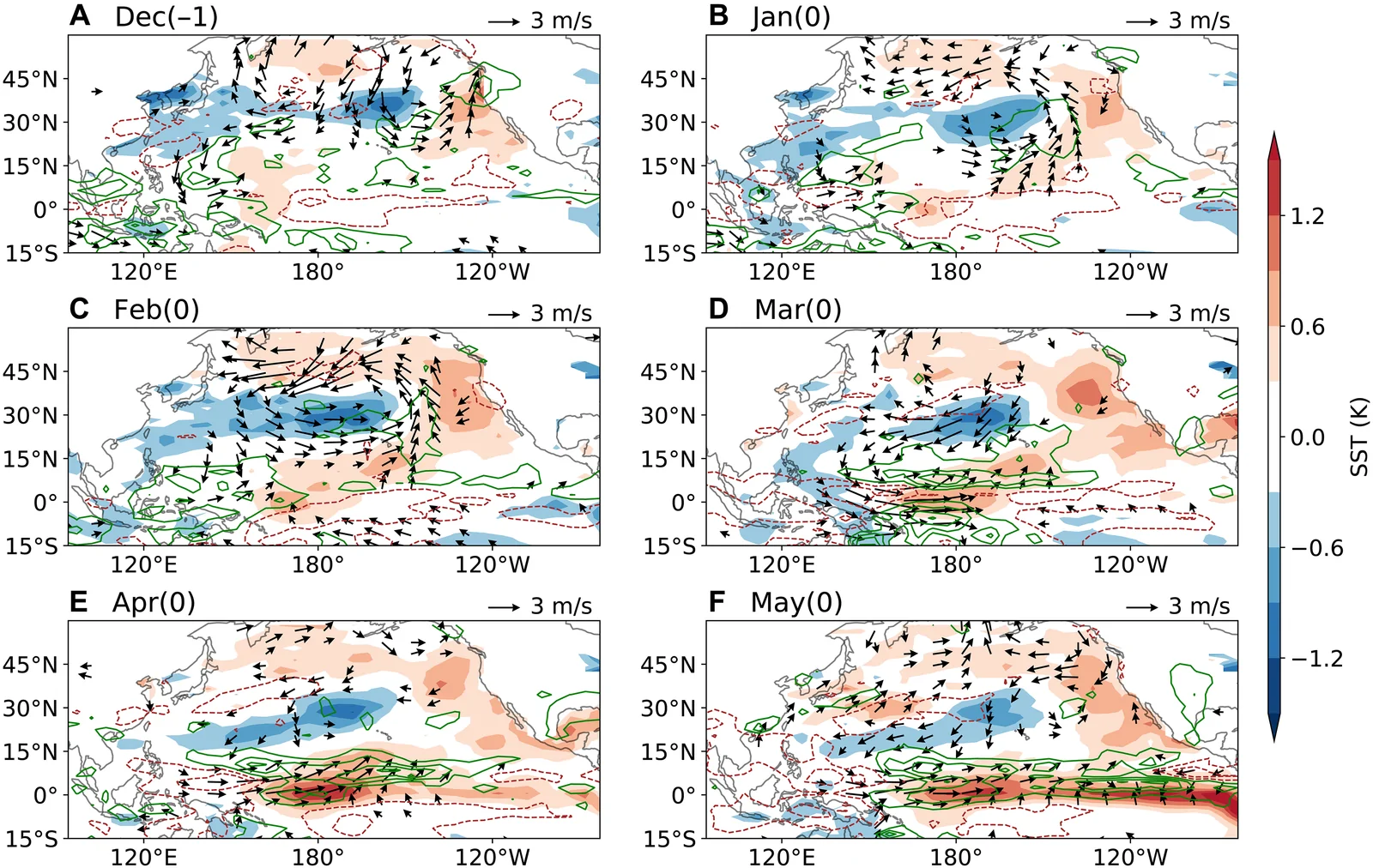
Horizontal patterns of ocean-atmosphere anomalies during the onset of the three extreme El Niño events. (**A** to **F**) Composite anomalies from December(−1) to May(0). Shading shows SST anomalies. Green and brown contours depict precipitation anomalies at 3-mm/day intervals, starting at 3 and −3 mm/day, respectively. Black arrows indicate surface wind anomalies; only vectors exceeding 1 m/s are displayed. Between December(−1) and February(0), East Asian cold surges, the North Pacific Oscillation, and the associated Pacific meridional mode are all present during each of the three extreme El Niño events.

Examining the horizontal SST anomaly structure of the three extreme El Niño events reveals that the warm western-central equatorial Pacific SST anomaly is connected to subtropical warm SST anomalies resembling the Pacific meridional mode (PMM) (*47*). These subtropical anomalies, induced by the North Pacific Oscillation (NPO) (*48*), appear consistently across all three events (Table 1) (*49*, *50*). From December(−1) to February(0), the subtropical warm SST anomalies propagate southwestward toward the equator, reinforcing the nascent equatorial warming in the western-central Pacific by March (Fig. 3D) (*43*, *51*, *52*).

**Table 1.** Normalized PMM and cold surge indices during the three extreme El Niño events. December(−1) to May(0) mean PMM indices and December(−1) to March(0) mean northerly cold surge indices (see Materials and Methods for definitions) for the 1982/1983, 1997/1998, and 2015/2016 events.

	1982/1983	1997/1998	2015/2016
PMM index	0.62	0.95	1.73
Cold surge index	1.76	2.43	0.98

Meanwhile, East Asian cold surges, characterized by anomalous northerlies over the western North Pacific and suppressed rainfall over Southeast China, are observed between December(−1) and March(0) in all three events (Fig. 3, A to D, and Table 1). These surges have previously been shown to induce WWBs in the western equatorial Pacific (*24*, *25*, *53*, *54*). As shown later, they also contribute to the initial development of the warm SST anomaly in the western-central equatorial Pacific.

Atmospheric model experiments reproduce the tropical wind anomalies associated with the coupled mode but fail to capture the subtropical circulation features (fig. S6). In particular, only weak cyclonic circulation anomalies west of North America are simulated in December(−1) and January(0), with amplitudes substantially smaller than observed. This discrepancy suggests that the NPO and cold surges arise primarily as stochastic atmospheric forcings (*52*), rather than as responses to the equatorial SST anomalies (*55*).

### Westerly wind–induced equatorial OHC increase

**Fig. 4.**
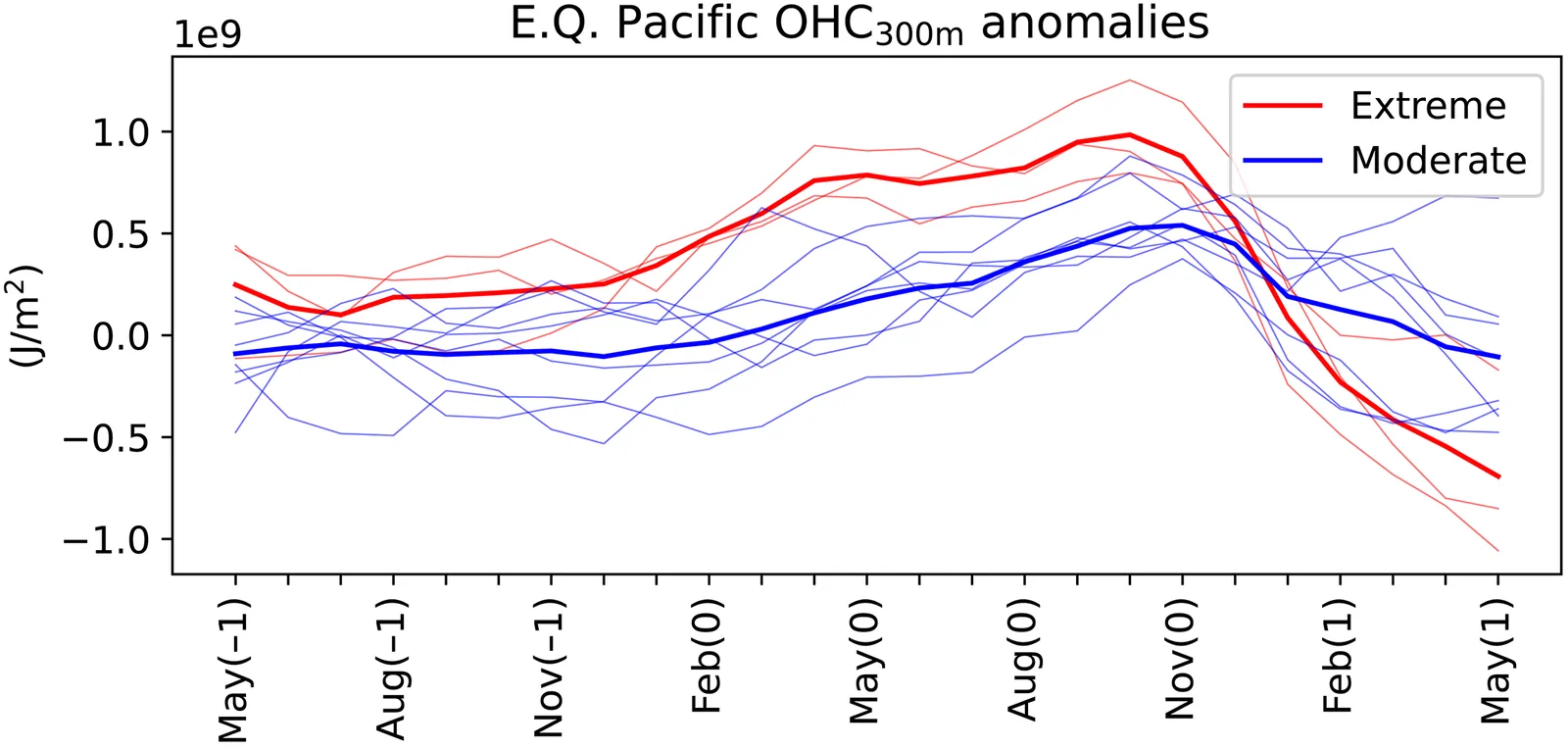
Temporal evolution of monthly equatorial upper ocean heat content during the three extreme El Niño events and eight moderate events. Thin lines show individual events, and thick red and blue lines denote the composites of the extreme and moderate events, respectively. Compared to the moderate events, the extreme events exhibit a faster and more consistent OHC increase from December(−1) to May(0).

**Fig. 5.**
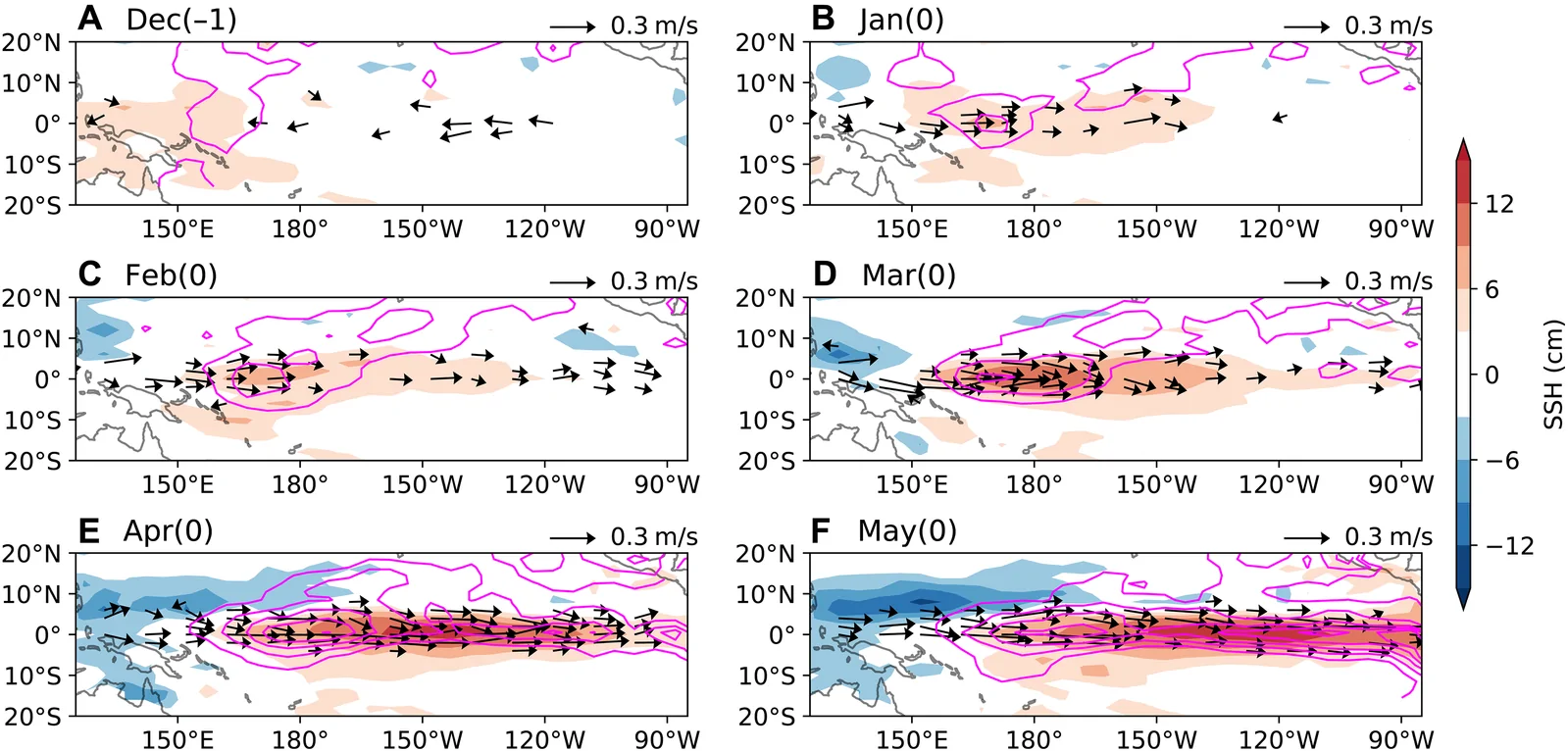
Equatorward transport of warm water during the extreme event onset. (**A** to **F**) Composite anomalies for the three extreme El Niño events from December(−1) to May(0). Shading shows sea surface height (SSH) anomalies. Black arrows indicate surface current anomalies averaged over the upper 50 m; only vectors exceeding 0.1 m/s are shown. Magenta contours represent positive SST anomalies at 0.3 K intervals, starting at 0.3 K. In April(0) and May(0), all three events exhibit similar upwelling Rossby waves in the subtropical western North Pacific and downwelling Kelvin waves in the equatorial central-eastern Pacific.

The equatorial westerly wind anomalies associated with the eastward-propagating coupled mode play a dominant role in building up the springtime equatorial OHC. Between December(−1) and May(0), the equatorial OHC more than triples—from about 2.5 × 10^8^ to 7.9 × 10^8^ J/m^2^ (Fig. 4). In December(−1), the equatorial Pacific is already in a recharged state, with OHC among the highest across El Niño events for the time (Fig. 4). It is characterized by elevated SSH in the western basin (Fig. 5A), likely resulting from oceanic adjustment to preceding easterly wind anomalies (*13*, *28*). As the onset coupled mode amplifies in the western-central equatorial Pacific, the westerly wind anomaly intensifies, driving equatorward Ekman transport of warm water from the off-equatorial western Pacific and suppressing equatorial cold-water upwelling (*26*, *28*). By May(0), the equatorial Pacific becomes fully recharged, characterized by elevated SSH and a deepened ocean thermocline in the central-eastern Pacific (Fig. 5F and fig. S5F). Meanwhile, positive wind stress curl along the poleward flank of the anomalous winds generates upwelling Rossby waves, discharging the western North Pacific (Fig. 5F). Because the westerly wind anomalies are displaced slightly north of the equator by the PMM-like SST anomalies (Fig. 3, C to E), the Rossby wave response is confined to the western North Pacific. The resulting zonal SSH gradients will ultimately cause equatorial OHC discharge through poleward pycnocline transport, as discussed in the ENSO recharge-discharge oscillator framework (*13*) and related studies (*28*, *29*).

While some previous studies have suggested that off-equatorial westerly wind anomalies linked to the PMM can recharge the ocean through downwelling Rossby waves (*56*), our results indicate that, for the three events considered here, the dominant pathway for the OHC increase between December(−1) and May(0) is equatorial westerly wind–forced downwelling Kelvin waves (*26*, *28*).

**Fig. 6.**
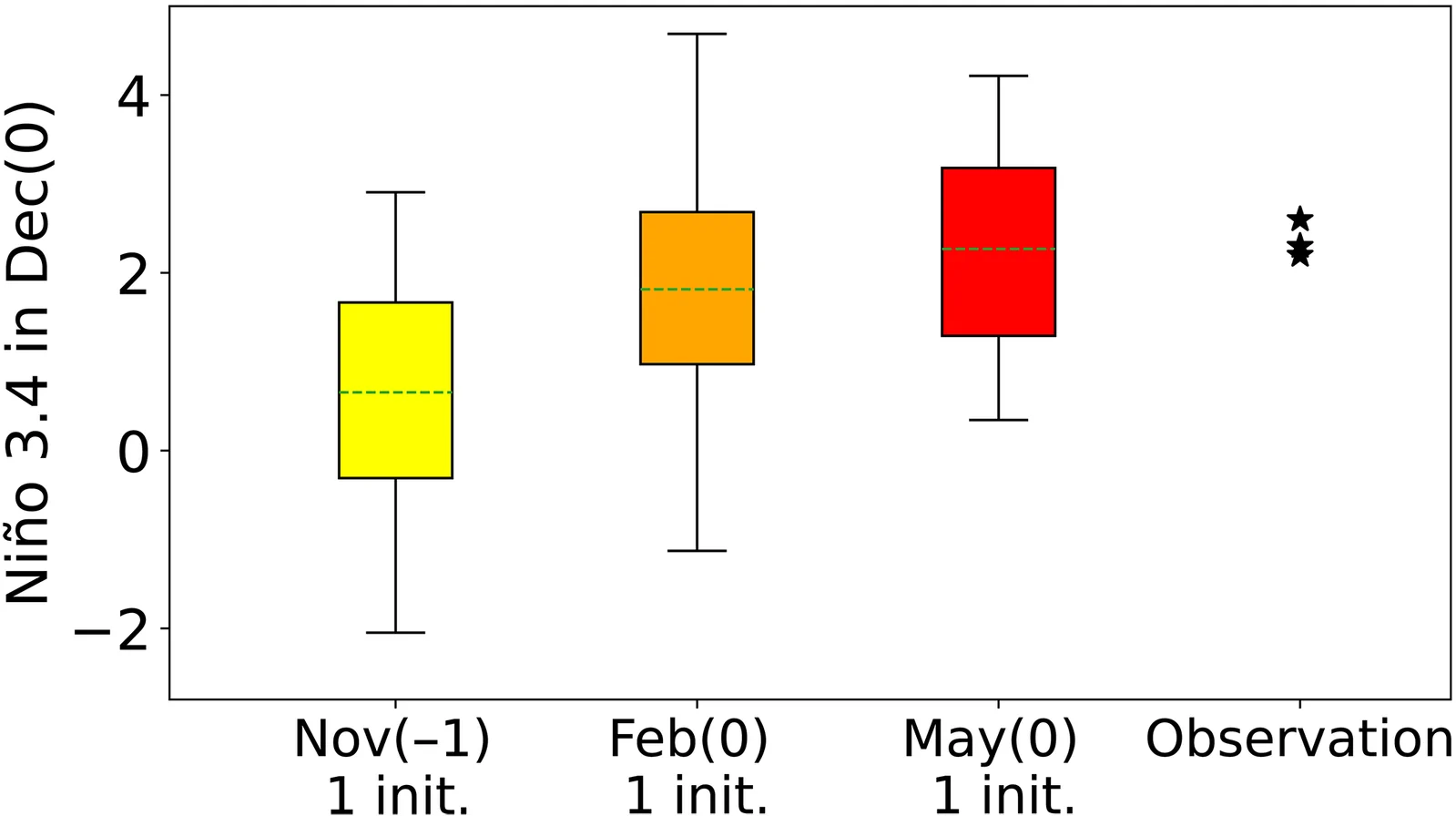
Ensemble hindcasts of the three extreme El Niño events. Boxplots show SMYLE reforecasts of the Niño 3.4 index in December(0), initialized on November(−1) 1 (yellow), February(0) 1 (orange), and May(0) 1 (red). Results from the three extreme events, each with 20 ensemble members (60 total), are combined. Green dashed lines denote the corresponding 60-member ensemble means, and three black stars mark the observed Niño 3.4 values. The ensemble-mean prediction increases substantially from the November(−1) initialization to the February(0) initialization.

The elevated OHC in May(0) preconditions the development of extreme El Niño events and, within the recharge oscillator framework, can be viewed as a Kelvin-wave response to westerly wind anomalies. In ensemble hindcasts initialized on May(0) 1 (Materials and Methods) and composited across the three extreme events, nearly all 60 ensemble members predict El Niño conditions by December(0), with an ensemble-mean Niño 3.4 index of 2.3 K and only one member falling below 0.5 K (Fig. 6). This is a substantial increase from the ensemble-mean prediction of 0.7 K in the November(−1) 1 initialized hindcasts. That earlier prediction reflects the contribution from recharge induced by prior-year easterly wind anomalies, whereas the additional 1.6 K increase largely reflects the impact of the westerly wind–induced OHC buildup. In contrast, May(0) 1 initialized hindcasts for the eight moderate events, which lack the onset coupled mode and the associated rapid OHC increase (Fig. 4), yield an ensemble-mean forecast of only 0.3 K, with little change from the November (−1) 1 initialization (fig. S7). These results therefore underscore the central role of the onset coupled mode in driving and predicting extreme El Niño events.

### Optimal stochastic wind forcings in winter

One notable feature in Fig. 6 is that the three extreme El Niño events become predictable as early as February(0). Composited across the events, hindcasts initialized on February(0) 1 already predict an ensemble-mean December(0) Niño 3.4 index of 1.8 K (signal-to-noise ratio = 1.54), exceeding the 1.5 K threshold for a major El Niño. However, 22 of the 60 members (37%) still fall below this threshold, indicating intrinsically limited predictability in boreal spring. Nevertheless, compared to the November(−1) 1 initialization that yields an ensemble mean of 0.7 K (signal-to-noise ratio = 0.52), this 1.1 K increase suggests that ocean-state changes driven by stochastic atmospheric variability between December(−1) and February(0) substantially enhance the events’ predictability.

**Fig. 7.**
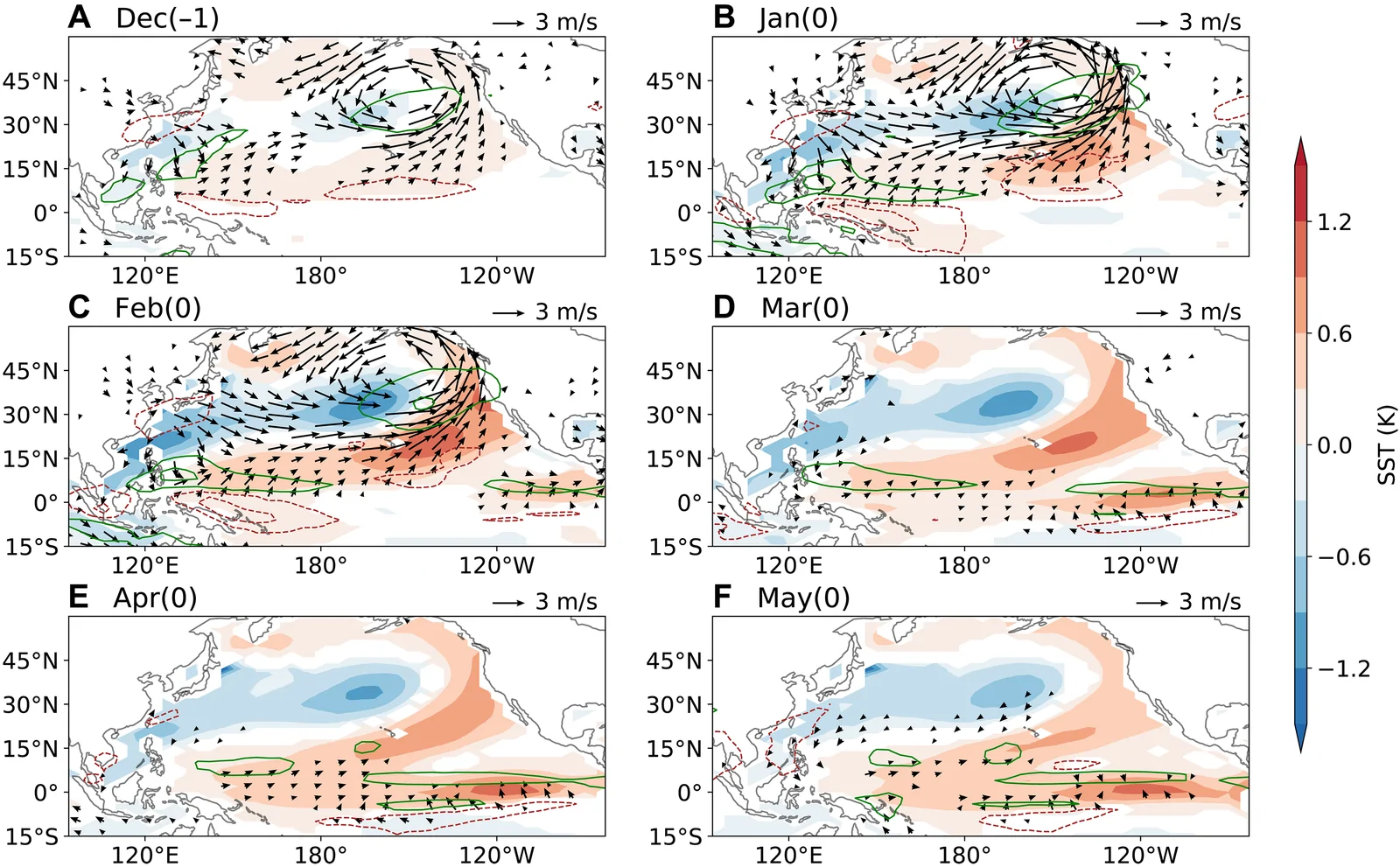
Cold surges and the NPO as optimal stochastic winter forcings in SMYLE hindcasts. (**A** to **C**) Optimal stochastic atmospheric forcing patterns during December(−1) to February(0) that most effectively enhance the Niño 3.4 index by December(0), based on the November(−1) 1 initialized hindcasts. Shading indicates the associated SST response. Green and brown contours denote precipitation anomalies at 3-mm/day intervals, starting from 3 and −3 mm/day, respectively. Arrows show surface wind anomalies; only vectors exceeding 0.5 m/s are displayed. (**D** to **F**) The subsequent coupled ocean-atmosphere evolution from March(0) to May(0). Only fields significant at the 95% confidence level are shown.

While the observational analysis is limited to the three extreme events, the ensemble hindcasts offer a much larger sample to diagnose the relevant stochastic processes. To isolate these processes, we analyze 1000 ensemble-spread samples (50 years × 20 members) from the November(−1) 1 hindcasts, treating each realization as independent (Materials and Methods). By regressing the February(0) SST spread onto the December(0) Niño 3.4 spread, we identify an optimal SST pattern that most effectively promotes subsequent El Niño growth. This pattern features a PMM-like warm SST anomaly in the subtropical Northeastern Pacific and a northwest-southeast SST dipole in the northwestern Pacific (*57*), resembling the observed anomalies (Fig. 3), in addition to a warm SST anomaly in the far-eastern equatorial Pacific (Fig. 7C). A similar structure has previously been identified as the “optimal initial SST perturbation” for ENSO development (*58*–*60*).

We further find that the northwestern Pacific and subtropical northeastern Pacific SST anomalies are induced by East Asian cold surges and the NPO, respectively, between December(−1) and February(0) (Fig. 7, A to C). The normalized seasonal-mean indices of the cold surges and the NPO are 1.31 and 0.84, respectively (Materials and Methods), and the two may be dynamically linked through mid-latitude Rossby waves (*61*). Cold surges induce westerly wind anomalies in the western equatorial Pacific, initiating the upper-ocean heat recharge through equatorward Ekman transport (figs. S8 and S9, A to C). The resulting weak warm SST anomalies then couple with the atmosphere and slowly propagate eastward (Fig. 7D and fig. S10A). By April(0), the southwestward-propagating PMM anomaly further reinforces the equatorial warming through the seasonal footprinting mechanism (Fig. 7D) (*43*, *52*, *62*), while the associated westerly wind anomalies continue to increase equatorial OHC, reaching a maximum value of 3.4 × 10^8^ J/m^2^ (fig. S8). Although westerly wind anomalies also emerge in the far-eastern equatorial Pacific in February(0), their contribution appears secondary, as OHC has already reached 2.4 × 10^8^ J/m^2^ by that time, with positive SSH anomalies concentrated in the western-central equatorial Pacific (fig. S9C).

Together, these results suggest that stochastic East Asian cold surges and NPO variability during boreal winter act as optimal subtropical forcings for triggering the onset coupled mode in the western-central equatorial Pacific and promoting equatorial OHC increase and subsequent El Niño development. In particular, they enable the three historical extreme events to partially overcome the spring predictability barrier. Whereas the seasonal footprinting mechanism implies that wintertime NPO forcing influences the equator primarily in late spring and summer (*52*, *62*), our results indicate that cold surges can induce equatorial westerly wind anomalies and OHC increase as early as December(−1).

It is also noteworthy that a warm SST anomaly is already present in the western equatorial Pacific in November(−1), before the onset of cold surges and the full establishment of the PMM (Fig. 1A). For the 1982/1983 and 1997/1998 events, this anomaly is likely associated with reflected downwelling Kelvin waves (fig. S1, D and E), whereas for 2015/2016, it is related to the preceding weak El Niño state. The 20-member ensemble mean initialized on November(−1) 1 captures the deterministic evolution of this early precursor (fig. S11). While it generates westerly wind anomalies and helps increase OHC during boreal spring, the wind anomalies are only 30 to 40% of observed strength (fig. S11A versus Fig. 1A), yielding a much weaker recharged ocean state by May(0) (fig. S11B versus Fig. 1B). Consequently, the composite December(0) Niño3.4 index reaches only 0.7 K.

## DISCUSSION

We have identified a slow, eastward-propagating coupled mode in the western-central equatorial Pacific during the early onset of El Niño conditions in boreal winter-spring as a critical factor in extreme El Niño events. In the CESM2 Large Ensemble, this mode precedes ∼80% of extreme El Niño events. It features slow eastward-propagating warm SST anomalies coupled with largely deterministic westerly wind anomalies, which intensify in spring through mutual interactions. Through equatorward Ekman transport, these winds drive a strong increase in equatorial OHC. In the 1982/1983, 1997/1998, and 2015/2016 events, equatorial OHC more than triples following this process, and hindcasts initialized on May(0) 1 yield an ensemble-mean December(0) Niño 3.4 index of 2.3 K, 1.6 K higher than in November(−1) 1 initializations. These results thus underscore the importance of this coupled mode for springtime OHC buildup and extreme El Niño onset.

We further show that East Asian cold surges and the NPO act as optimal winter stochastic forcings that can trigger or amplify this onset coupled mode. In doing so, they enable early prediction of the three extreme events and help partially overcome the spring predictability barrier. Although cold surges and the NPO have long been linked to a broad range of El Niño events (*58*), particularly Central Pacific (CP)–type events via the NPO-induced PMM (*63*, *64*), this does not contradict our findings. The 1982/1983, 1997/1998, and especially 2015/2016 events all exhibit pronounced CP warming (fig. S1, A to C). Even the 1982/1983 and 1997/1998 events, typically classified as Eastern Pacific–type events, have C-indices (used to measure the amplitude of CP-type El Niño) comparable to composite CP events (*65*). Our results therefore reconcile the role of subtropical forcings with the occurrence of extreme El Niño events.

Winter cold surges and NPO do not guarantee extreme El Niño events, as illustrated by the failed 2014/2015 event (*66*). Although the February(0) 1 initialized hindcasts predict an ensemble-mean December(0) Niño 3.4 index of 1.8 K for the three extreme events, the ensemble spread remains large. Even for the May(0) 1 initializations, 22 of the 60 members remain below 2 K, suggesting that stochastic processes in the following summer and fall continue to modulate outcomes. Previous studies have found that summertime easterly wind bursts or weak air-sea coupling could disrupt El Niño growth and lead to the failure of extremes (*16*, *17*, *67*). Future analysis of the May(0) 1 initialized ensemble hindcasts may help reveal key summertime processes controlling success versus failure.

Together, our findings suggest that improving the representation and assimilation of western-central Pacific air-sea coupling and extratropical circulation variability in climate models is essential for enhancing early warnings of extreme El Niño events—an urgent goal given their profound global impacts and projected intensification under future climate change (*68*–*70*).

## MATERIALS AND METHODS

### Datasets and model experiments

We analyze El Niño events using the ERA5 atmospheric reanalysis (*71*) and ORAS5 ocean reanalysis (*72*) for the period 1980 to 2019. For the CESM2 Large Ensemble, we use 10 ensemble members branched from model year 1231 of the preindustrial control simulation and subsequently integrated under historical and future forcings, including smoothed CMIP6 biomass-burning emissions (*73*). The simulations span 1850 to 2100, and our analysis focuses on 1980 to 2019.

AGCM experiments are conducted using NeuralGCM at 2.8° resolution, integrated from 1980 to 2020 (*74*); 28 ensemble members that run stably across the entire period are used. To assess El Niño predictability and identify optimal winter atmospheric forcings, we use the Seasonal-to-Multiyear Large Ensemble (SMYLE) hindcasts generated with the CESM2 (*75*). CESM2 uses a 1° ocean model (POP2) coupled to a 1° atmosphere model (CAM6-FV). SMYLE provides 20-member ensembles initialized quarterly from 1970 to 2019; here, we analyze hindcasts initialized on November 1, February 1, and May 1.

To isolate interannual El Niño variability, we first linearly detrend the reanalysis and CESM2 Large Ensemble data and then remove the climatological seasonal cycle. For SMYLE hindcasts, we first subtract a centered 10-year running mean, followed by removal of the climatological seasonal cycle.

### Extreme and moderate El Niño years

We define year (0) as the El Niño developing year, with years (−1) and (1) denoting the preceding and following years, respectively. For the three historical extreme El Niño events, the mean SST anomaly over 160°E to 160°W, 5°N to 5°S during December(−1) to May(0) is 0.53 K. This region encompasses the warm western-central Pacific SST anomaly that consistently precedes the three extreme events. For comparison with the extremes, we select El Niño events in which SST anomalies in this region remain below 0.53 K in every month from December(−1) to May(0). Eight events satisfy this criterion: 1986/1987, 1994/1995, 2002/2003, 2004/2005, 2006/2007, 2009/2010, 2014/2015, and 2018/2019. Among these, only 2009/2010 qualifies as a major event [Niño 3.4 > 1.5 K in December(0)]. We thus refer to this group collectively as moderate events.

In the CESM2 Large Ensemble, El Niño events are defined when the November(0)-December(0)-January (1) (NDJ) mean Niño 3.4 index exceeds 0.5 SDs (0.83 K). Extreme El Niño events are defined when the NDJ Niño 3.4 index exceeds 1.5 SDs (2.48 K). Across the 10 ensemble members during 1980 to 2019, 132 El Niño events are identified, including 34 extreme events; the remainder are classified as moderate. For the 34 extreme events, the mean December(−1) to May(0) SST anomaly over 160°E to 160°W, 5°N to 5°S is 0.79 K. Among them, 27 events (79%) are preceded by a warm SST anomaly in this region exceeding 0.79 K in at least 1 month during December(−1) to May(0).

### Mixed layer heat budget analysis

To reveal the growing mechanisms of the eastward-propagating warm SST anomaly in the western-central equatorial Pacific between November(−1) and May(0), we conduct a mixed layer heat budget analysis following Liang *et al.* (*43*), with a mixed layer depth set to 50 m. The governing equation can be written as

$$\begin{array}{c} \partial T^{\prime }/\partial t={\left(-u\partial T/\partial x\right)}^{\prime }-{\left(v\partial T/\partial y\right)}^{\prime }-{\left(w\partial T/\partial z\right)}^{\prime }+\\ \left({\text{SW}}^{\prime }+{\text{LW}}^{\prime }+{\text{LHF}}^{\prime }+{\text{SHF}}^{\prime }\right)/c_{p}\rho H \end{array}$$ (1)

where *T* is ocean temperature; *u*, *v*, and *w* are zonal, meridional, and vertical velocities, respectively; SW and LW represent net surface shortwave and longwave fluxes; and LHF and SHF represent latent and sensible heat fluxes. $c_{p}$ is the specific heat capacity of seawater, set at 3990 J kg^−1^ K^−1^; ρ is seawater density, set at 1025 kg/m^3^; *H* is the mixed layer depth. A prime (′) indicates interannual anomalies.

### Definition of PMM, cold surge, and NPO indices

The monthly PMM SST index is obtained from NOAA PSL https://psl.noaa.gov/data/timeseries/month/PMM/. For each year, we compute the December(−1) to May(0) mean of the monthly PMM index and normalize it to quantify the average PMM strength during boreal winter-spring.

Cold surges during December(−1) to March(0) are identified on a daily basis. A cold surge is defined to occur when daily 10-m northerly wind anomalies averaged over 120°E to 140°E, 10°N to 20°N exceed 1 SD (2.0 m/s), following Feng *et al.* (*53*). The climatological northerly wind speed in this region during December to March is 3.2 m/s. We quantify the accumulated cold surge strength in each winter by summing the daily northerly wind anomalies associated with all identified cold surge events and normalizing the resulting seasonal total.

The cold-surge strength shown in Fig. 7 is measured using monthly data. Specifically, we calculate the December(−1) to February(0) mean northerly wind anomalies over 120°E to 140°E, 10°N to 20°N, and normalize them by the SD of seasonal-mean northerly wind speed over the same region, computed from the SMYLE November (−1) 1 initializations. The NPO is defined as the second EOF of sea level pressure (SLP) anomalies over 110°E to 90°W, 15°N to 75°N, following Liang *et al.* (*43*). The seasonal-mean NPO strength is then obtained by regressing the mean SLP anomalies shown in Fig. 7 (A to C) onto the NPO-related SLP pattern.

### Identification of optimal stochastic winter wind forcings

We use November(−1) 1 initialized 20-member ensemble hindcasts for all 50 years to isolate the stochastic winter wind forcings that amplify subsequent El Niño growth. Following Liang *et al.* (*43*), we form ensemble-spread fields for each year and member by subtracting the 20-member mean from each member for precipitation, surface winds, and SST. To extract the SST structure most conducive to subsequent El Niño growth, we regress the February(0) SST spread (90°E to 80°W, 30°N to 10°S) onto the December(0) Niño 3.4 spread across all samples (50 years × 20 members = 1000). February(0) is chosen because the ensemble-mean prediction of December(0) Niño 3.4 increases from 0.7 K in the November(−1) 1 initializations to 1.8 K in the February(0) 1 initializations for the three extreme El Niño events. The resulting regression pattern defines the optimal February(0) SST forcing.

We then project each member’s February(0) SST-spread field onto this pattern to obtain an amplitude series (1000 values). Further regressing December(−1) to February(0) precipitation and wind spread fields onto this amplitude series yields corresponding optimal stochastic winter atmospheric forcings that excite this SST pattern. Regressions of March(0) to May(0) SST spread (and the spread of other variables) onto the same series diagnose the subsequent air-sea evolution (Fig. 7). Signals in November(−1) are negligible, as the initial ensemble spread is small due to the ocean-atmosphere memory. For comparability, all regression maps are rescaled to a + 1 K change in the December(0) Niño 3.4 index.

### Statistical analysis

For the composite of the three extreme El Niño events in Fig. 1, we retain only anomalies that share the same sign across all three events. Under a null hypothesis of random sign (with equal probability of positive and negative anomalies), the probability that all three events exhibit the same sign is 0.25. For the composite of eight moderate El Niño events (fig. S2), we perform a one-sample permutation test with sign-flip against 0 using all 2^8^ = 256 flips (*P* = 0.1). Statistical significance of the composite anomalies in Fig. 2 is assessed using a two-sided Student’s *t* test (*P =* 0.05). Regression coefficients in Fig. 7 are also evaluated with two-sided *t* tests (*N* = 1000; *P* = 0.05).
