# Supplementary material for: An eastward-propagating coupled mode amplifying the early growth of extreme El Niño events
Source: Sci Adv. 2026 Sep 23;12(39):eaee0444. doi: 10.1126/sciadv.aee0444 (PMC13600214; doi:10.1126/sciadv.aee0444)
Supplement: Supplementary file 1 [file sciadv.aee0444_sm.pdf]

Science Advances
AAAS

Supplementary Materials for

An eastward-propagating coupled mode amplifying the early growth of extreme El Niño events

Yu Liang *et al.*

Corresponding author: Yu Liang, yul257@ucsd.edu; Shang-Ping Xie, sxie@ucsd.edu

Sci. Adv. **12**, eaee0444 (2026)
DOI: 10.1126/sciadv.aee0444

This PDF file includes:

Figs. S1 to S11

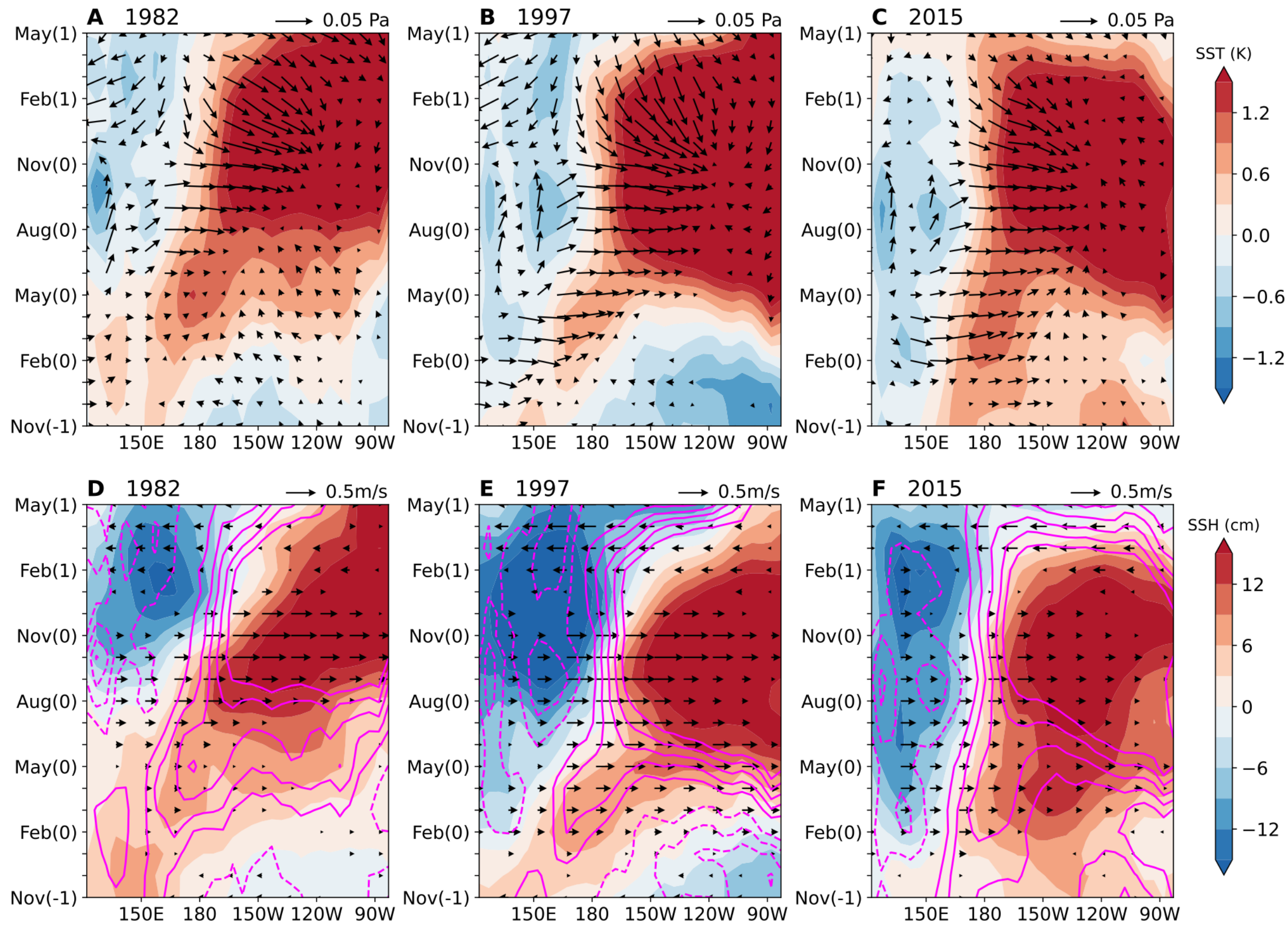

Fig. S1. Spatiotemporal evolution of individual extreme El Niño events. (**A-C**) Hovmöller diagrams of SST anomalies (shading) and wind stress anomalies (arrows) for the 1982/83, 1997/98, and 2015/16 events, respectively. All three events exhibit an eastward-propagating warm SST anomaly in the western-central equatorial Pacific during boreal winter and spring preceding major El Niño development. (**D-F**) As in (**A-C**), but for sea surface height anomalies (SSH; shading), SST anomalies (contours), and zonal current speed anomalies averaged over the upper 50m (arrows). Anomalies are equatorially averaged (5°N-5°S) and a 3-month running mean is applied. Only vectors exceeding 0.005 Pa in (**A-C**) and 0.05 m/s in (**D-F**) are shown.

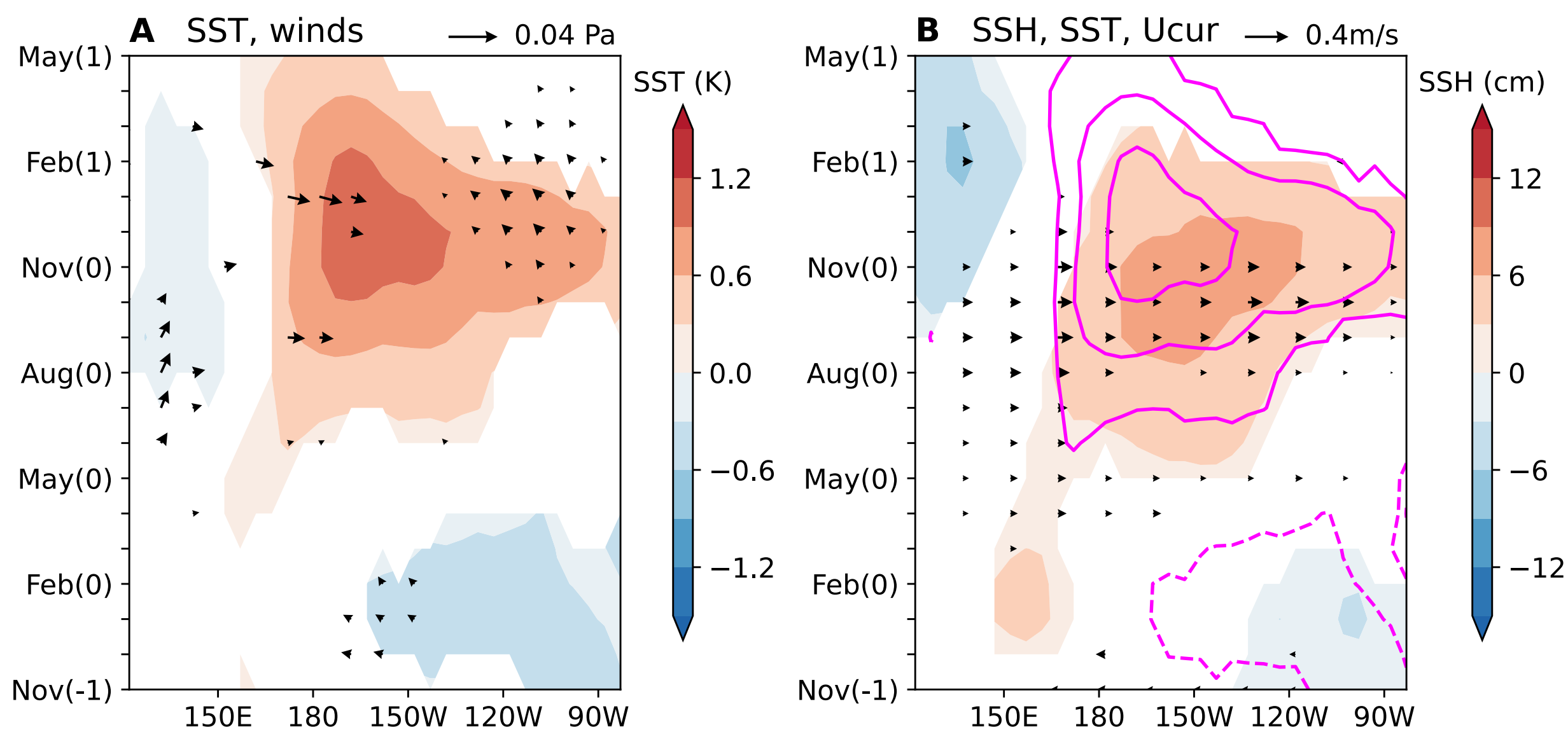

Fig. S2. Spatiotemporal evolution of eight moderate El Niño events. (A) Hovmöller diagram of SST anomalies (shading) and wind stress anomalies (arrows), composited for the 1986/87, 1994/95, 2002/03, 2004/05, 2006/07, 2009/10, 2014/15, and 2018/19 events, which are not preceded by strong warm SST anomalies in the western-central equatorial Pacific between December(-1) and May(0). **(B)** Same as in **A**, but for sea surface height anomalies (SSH; shading), SST anomalies (contours), and zonal current speed anomalies averaged over the upper 50m (arrows). Anomalies are averaged within the equatorial band (5°N-5°S) and a 3-month running mean is applied.

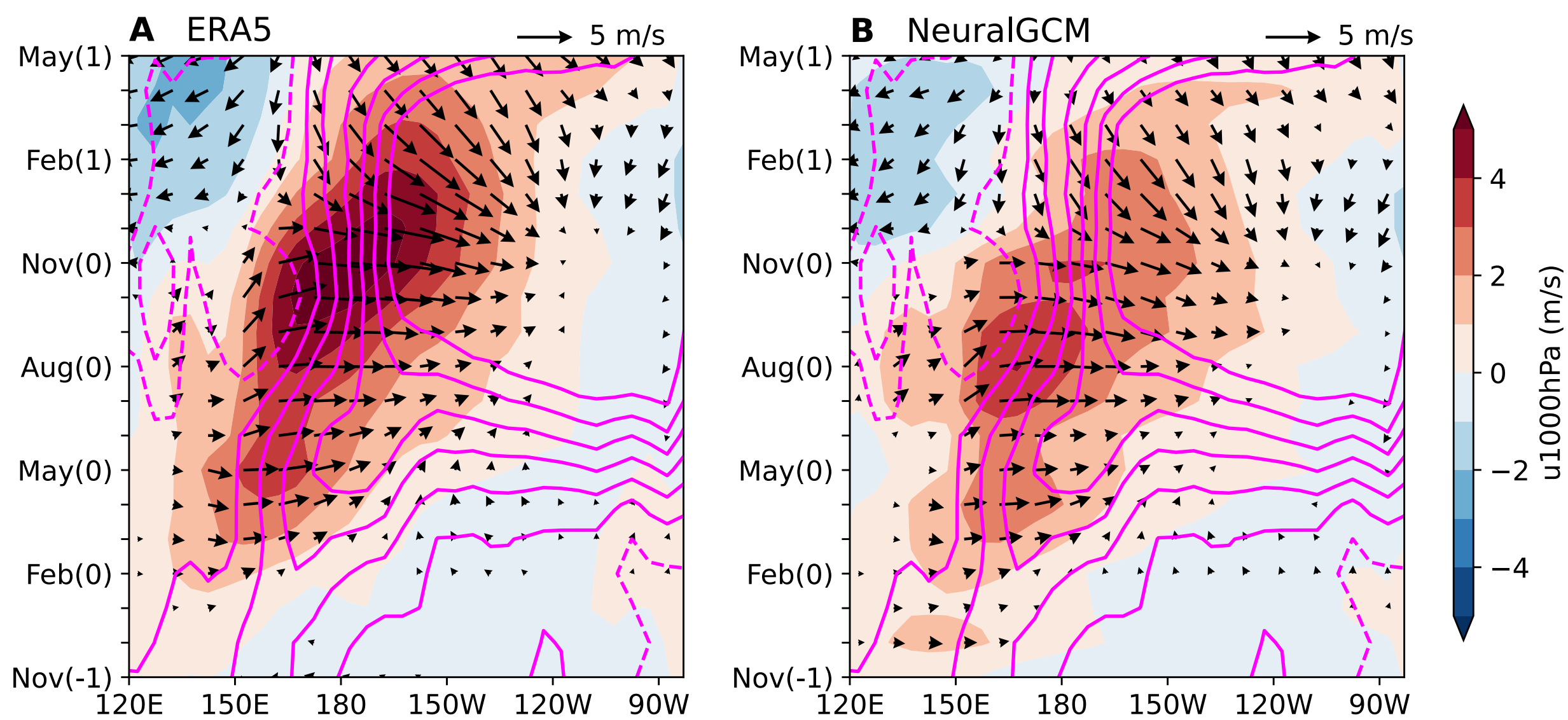

Fig. S3. Equatorial westerly wind anomalies in ERA5 and AGCM simulations. (**A**) Equatorially averaged (5°N-5°S) zonal wind anomalies, smoothed using a 3-month running mean and composited for the three extreme El Niño events (shading). Arrows denote equatorially averaged horizontal wind anomalies; only vectors exceeding 0.5 m/s are displayed. Magenta contours show the equatorially averaged SST anomalies at 0.3 K contour intervals, with solid contours starting at 0 K and dashed contours starting at -0.3 K. (**B**) Same composite zonal wind anomalies simulated by NeuralGCM. Simulations by CAM6 yield similar results.

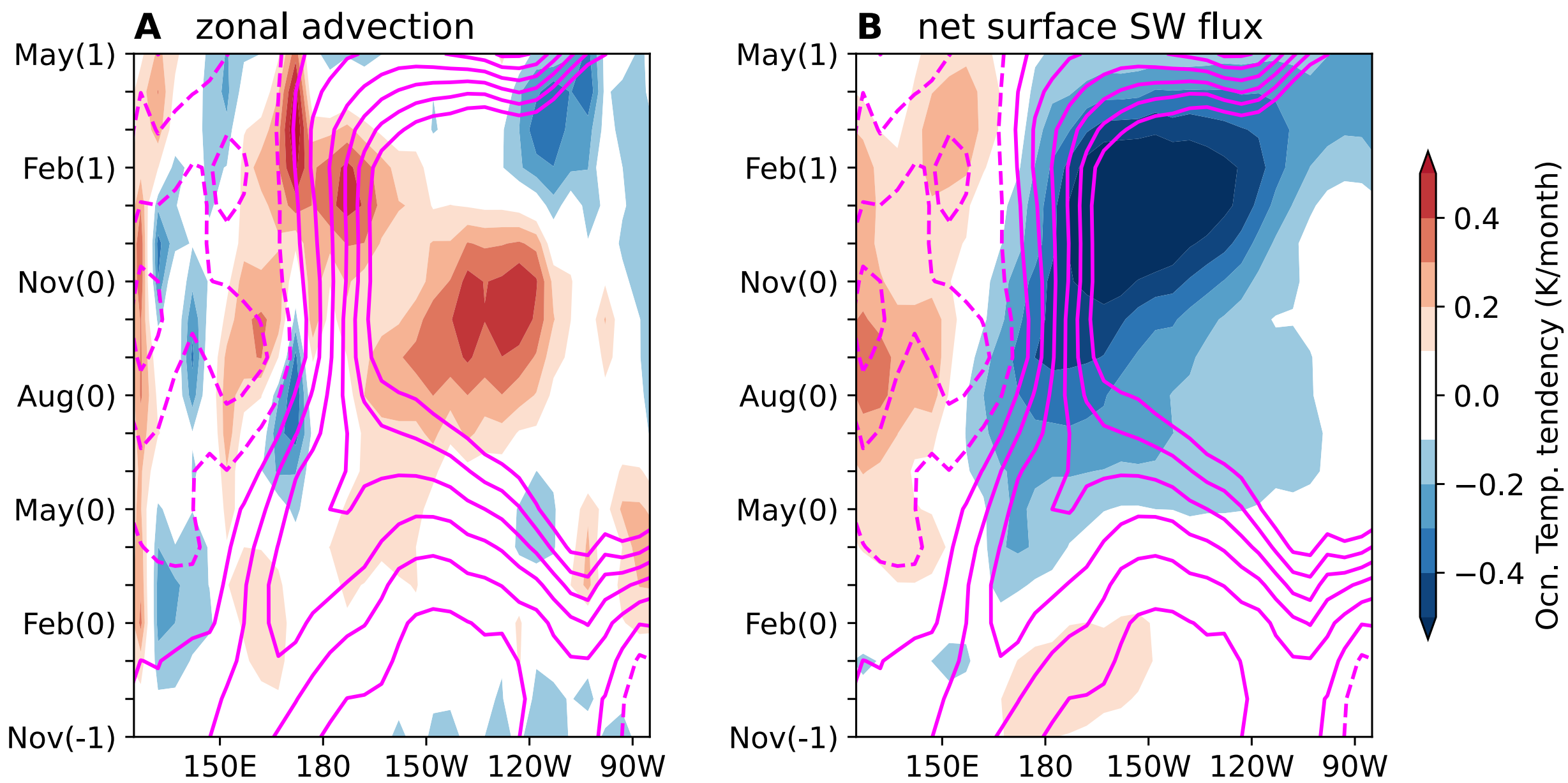

Fig. S4. Mixed-layer heat budgets of the onset coupled mode in the western-central equatorial Pacific. (**A**) Hovmöller diagram of zonal heat advection anomalies (shading), composited for the three extreme El Niño events. Magenta contours denote concurrent upper-50m ocean temperature anomalies. (**B**) Net surface shortwave flux anomalies. Anomalies are equatorially averaged (5°N-5°S) and a 3-month running mean is applied. Zonal advection provides the dominant positive feedback for the growth of the warm anomaly, while shortwave flux initially contributes to but later damps it.

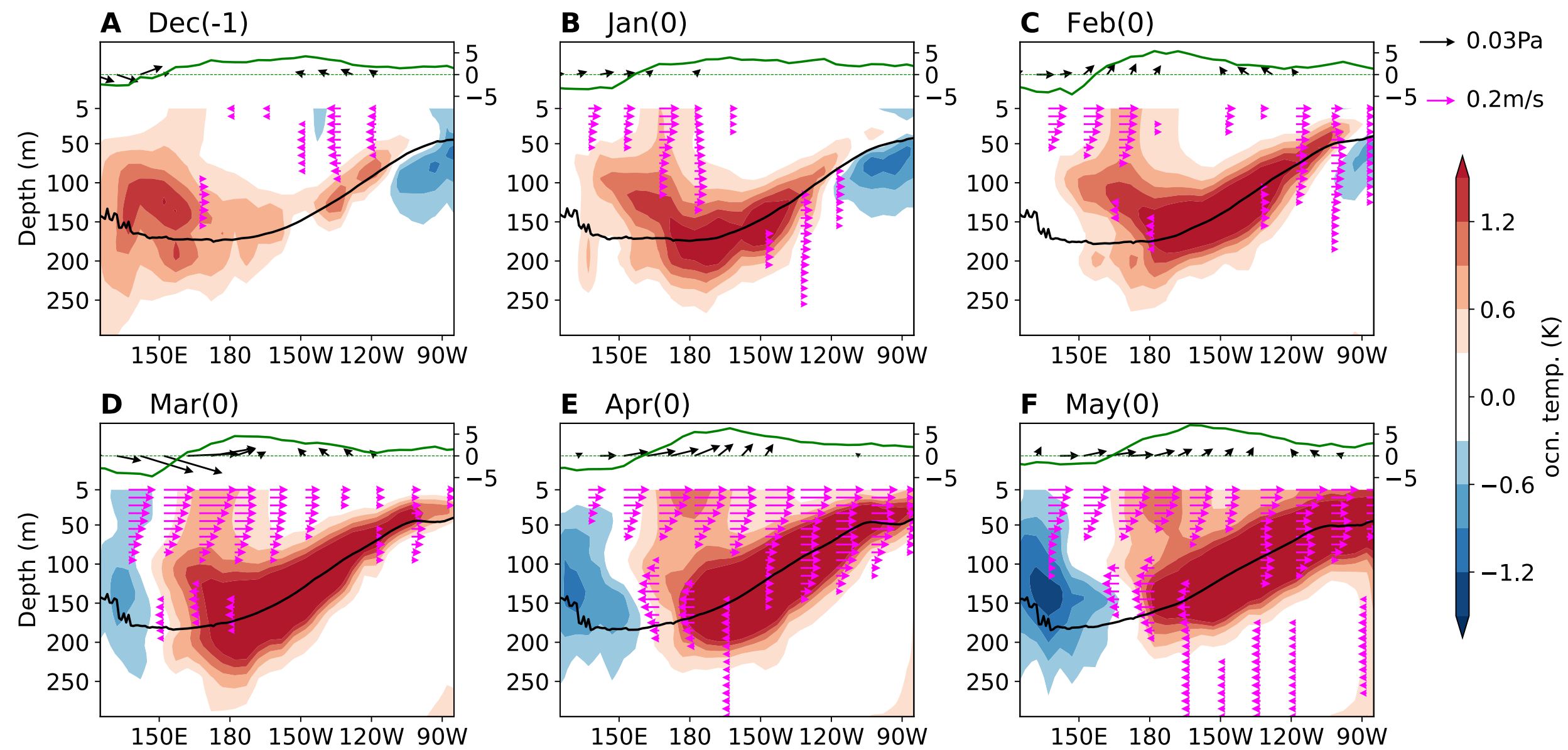

Fig. S5. Anomalies of equatorially averaged surface wind stress, precipitation, ocean temperature and zonal current speed. (**A–F**) Equatorially averaged (5°N-5°S) surface wind stress anomalies (black arrows), precipitation anomalies (green line), subsurface temperature anomalies (shading), and zonal current speed anomalies (magenta arrows) from December(−1) to May(0), composited for the three extreme El Niño events. Only wind stress anomalies exceeding 0.005 Pa and zonal current anomalies exceeding 0.05 m/s are shown. Black lines denote the monthly climatological 20°C isotherm.

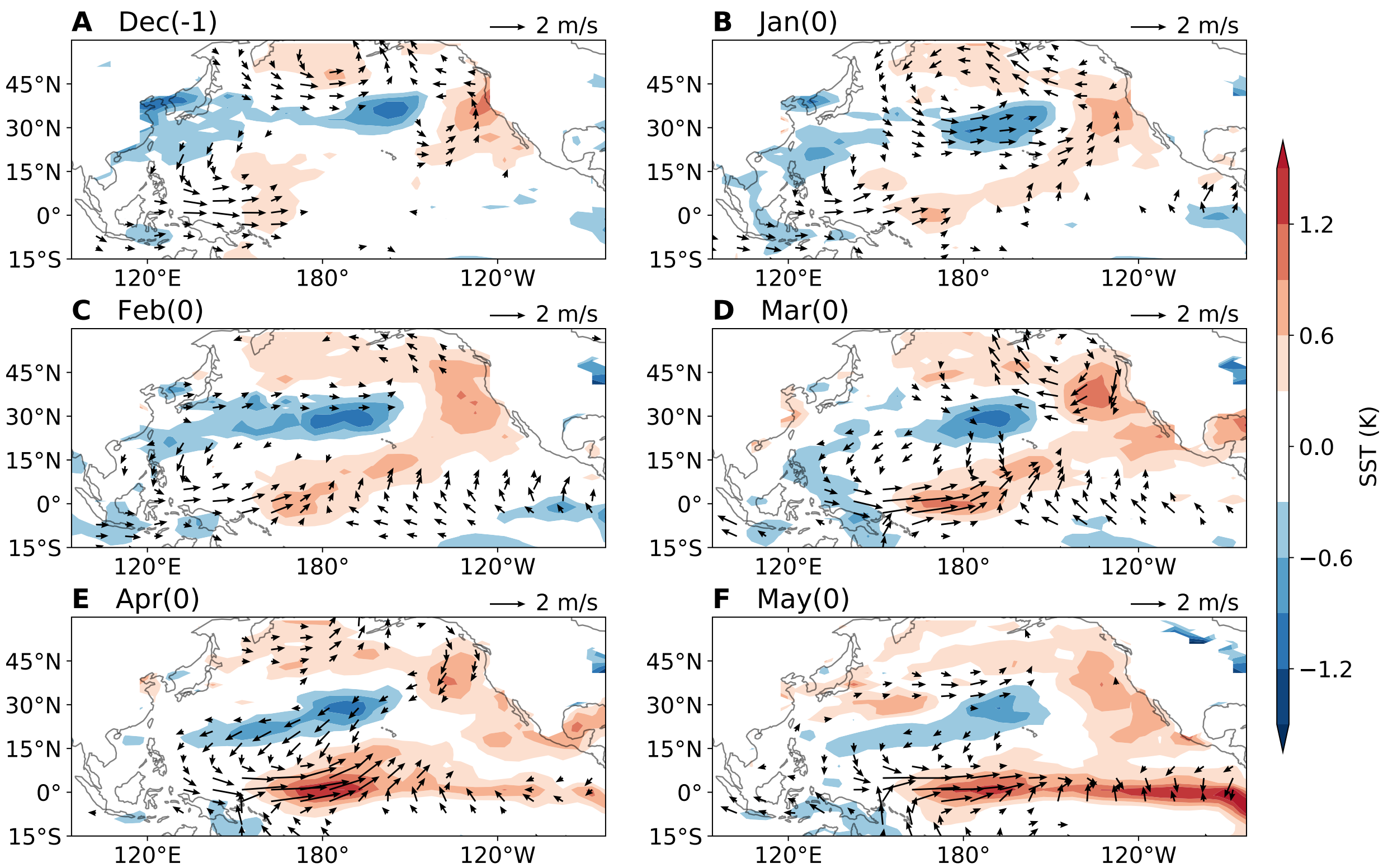

Fig. S6. Horizontal patterns of surface wind anomalies simulated by NeuralGCM. (**A–F**) Composite anomalies of surface winds (arrows) and SST (shading) from December(−1) to May(0). Only wind vectors exceeding 0.2m/s are shown. NeuralGCM reproduces the observed tropical wind anomaly pattern well (cf. Fig. 3).

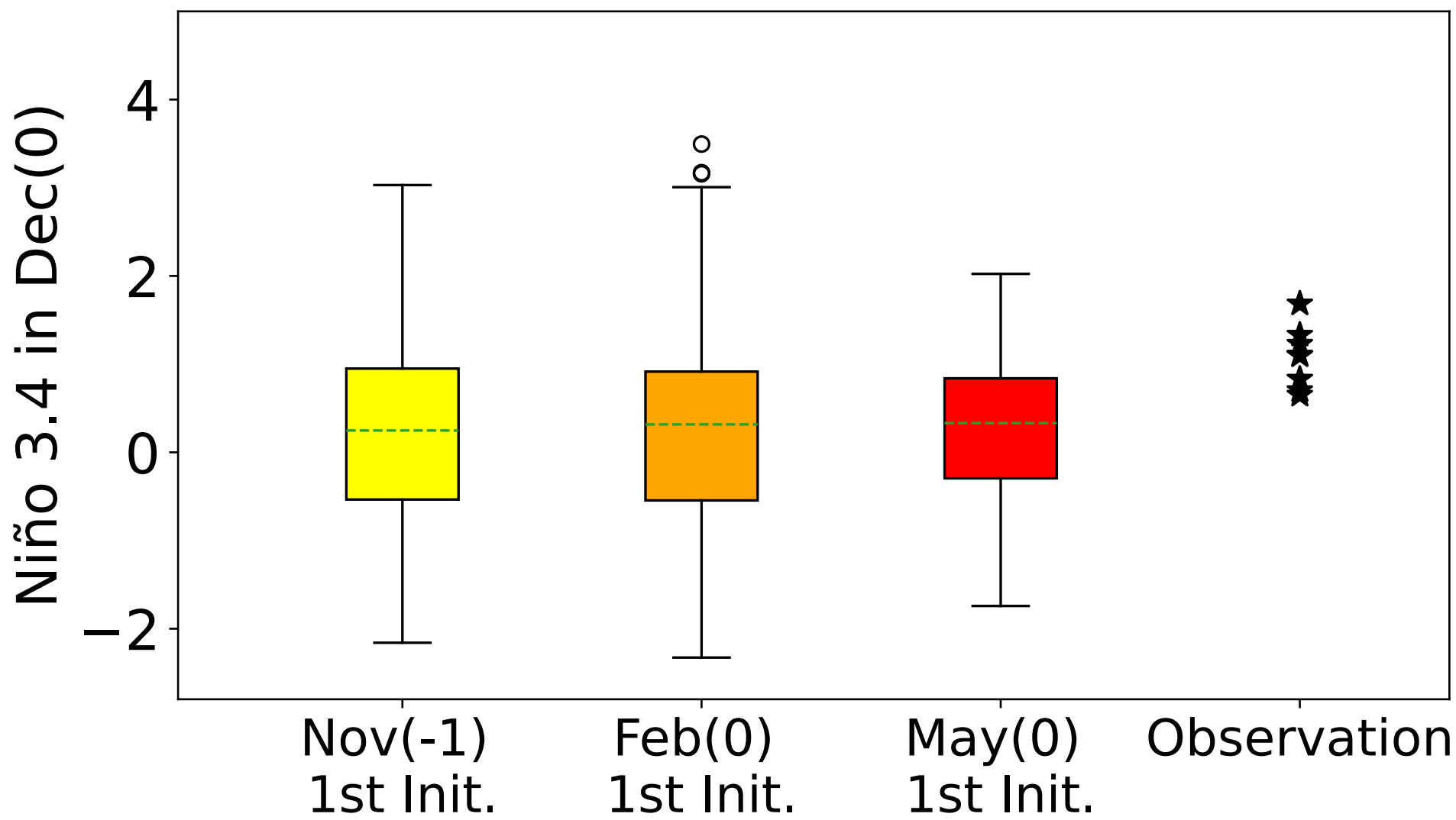

Fig. S7. Ensemble hindcasts of the eight moderate El Niño events. Same as Fig. 6, but for the eight moderate events. Hindcasts initialized on May(0) 1st yield an ensemble-mean Niño 3.4 index of only 0.3 K, suggesting little to no predictive signal.

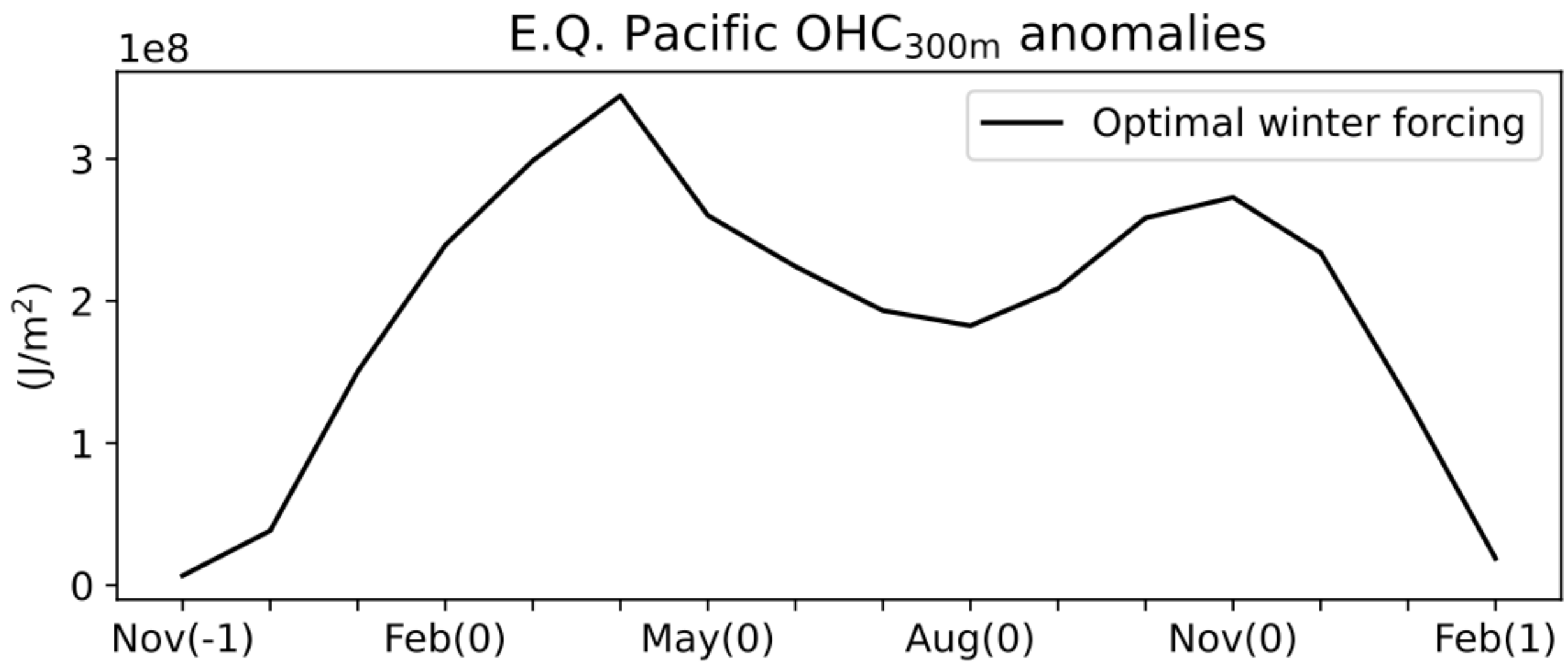

Fig. S8. Temporal evolution of equatorial OHC associated with the optimal stochastic winter atmospheric forcings. The result is derived from the ensemble spread of SMYLE hindcasts initialized on November(–1) 1st.

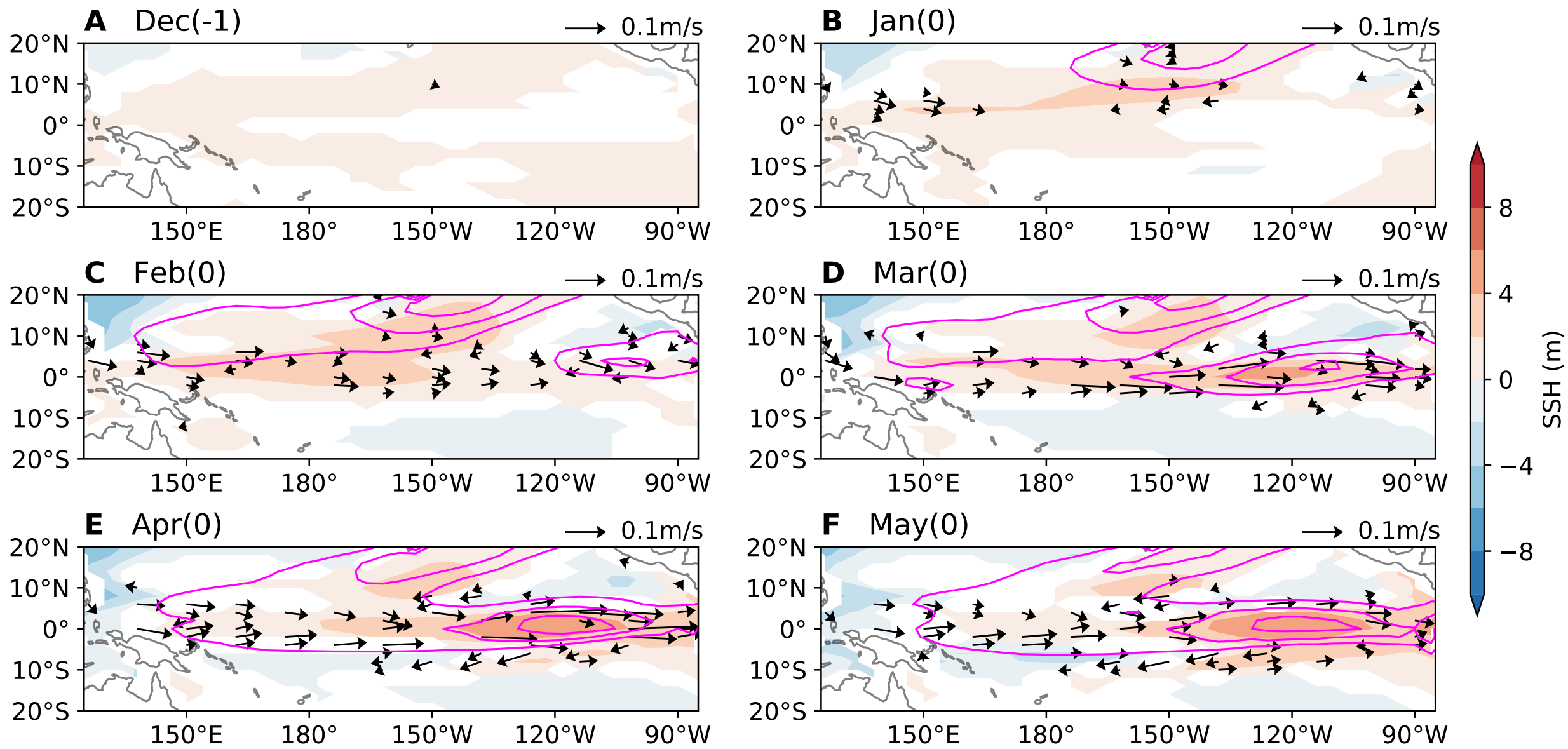

Fig. S9. Equatorial OHC recharge driven by the optimal stochastic winter forcings. (**A–C**) Sea surface height (SSH) anomalies driven by the East Asian cold surges and NPO between December(–1) and February(0). Magenta contours show concurrent positive SST anomalies at 0.3 K intervals, starting at 0.3 K. Black arrows represent ocean current anomalies averaged over the upper 50 m. (**D–F**) Subsequent ocean evolution from March(0) to May(0). Only fields significant at the 95% confidence level and ocean current vectors exceeding 0.02 m/s are shown.

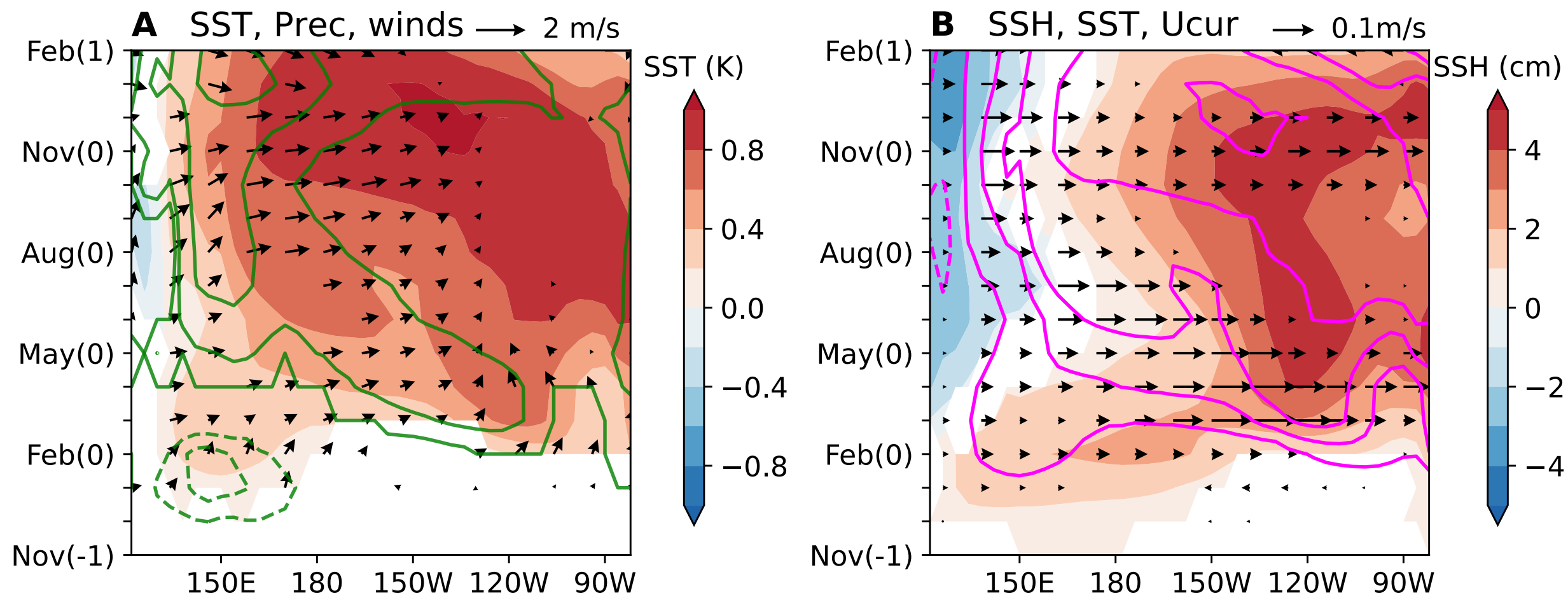

Fig. S10. Equatorial ocean-atmosphere evolution following the optimal stochastic winter forcings. (**A**) Equatorially averaged (5°N-5°S) SST (shading), precipitation (contours; 1 mm/day intervals, solid ≥ 0, dashed ≤ -1), and surface wind (arrows) anomalies following the East Asian cold surges and NPO in December(–1)-February(0). (**B**) Equatorially averaged SSH anomalies (shading) and upper-50 m zonal current anomalies (arrows); magenta contours denote concurrent SST anomalies. A slow, eastward-propagating SST anomaly similar to the observation can be observed in the western-central equatorial Pacific between January(0) and May(0), preceding the subsequent El Niño development.

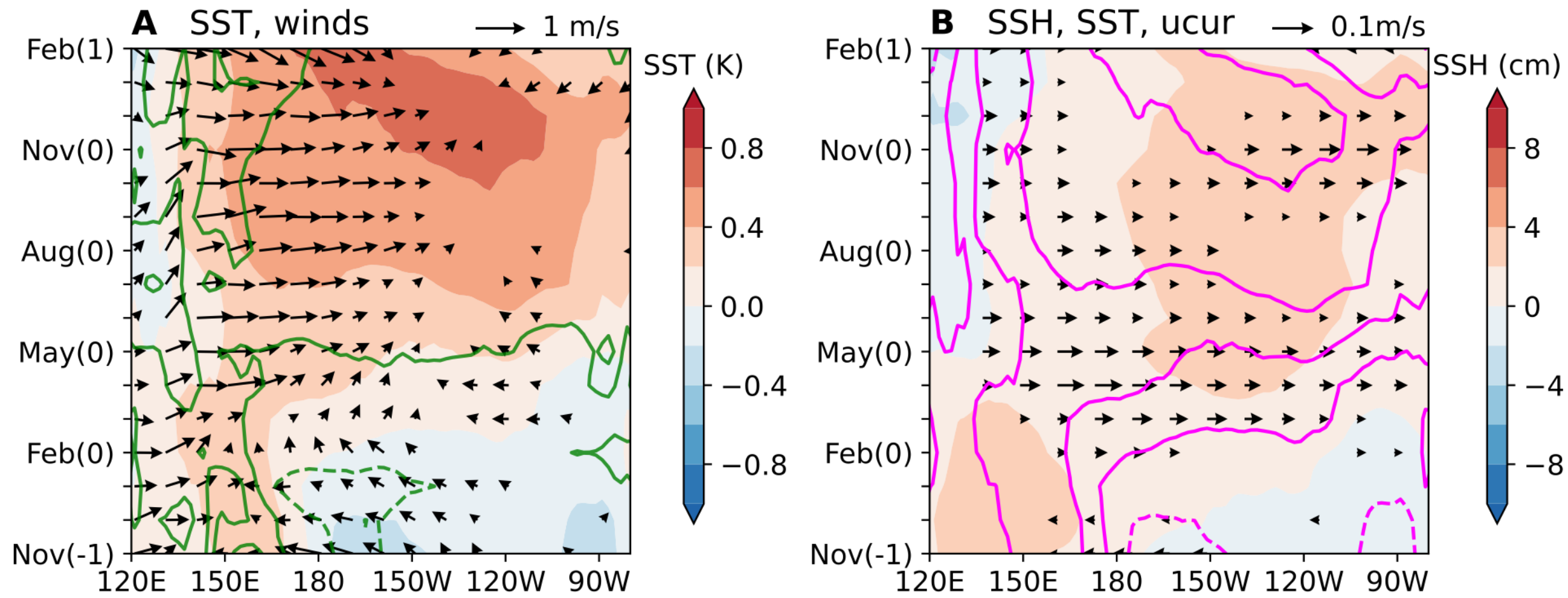

Fig. S11. Ensemble-mean evolution of hindcasts initialized on November(–1) 1st, composited for the 1982/83, 1997/98, and 2015/16 events. (**A**) Equatorially averaged (5°N-5°S) SST (shading), precipitation (contours; 1 mm/ day intervals, solid ≥ 0, dashed ≤ –1), and surface wind (arrows) anomalies. (**B**) Equatorially averaged SSH anomalies (shading) and upper-50 m zonal current anomalies (arrows); magenta contours denote concurrent SST anomalies. Only wind vectors exceeding 0.2 m/s in (**A**) and zonal current vectors exceeding 0.02 m/s in (**B**) are shown. Between November(–1) and May(0), equatorial SST, westerly wind, and SSH anomalies reach only 30–40% of the observed amplitudes shown in Fig. 1.
